# XXVIII Reunión Anual de la Sociedad Canaria de Neurología (SOCANE). Libro de Comunicaciones

**DOI:** 10.31083/RN55097

**Published:** 2026-08-25

## 1. Comunicaciones Orales

**CO-01.- Talamotomía Mediante Ultrasonidos Focalizados (HIFU) en Temblor Esencial: Experiencia Actualizada en el Hospital Universitario de Gran Canaria Doctor Negrín**

Jorge Cegarra Sánchez^1^, Juan Diego Guerra Hiraldo^1^, Juan Francisco García Granado^1^, Alejandro Relloso de la Fuente^1^, Alicia Auyanet Matejkova^1^, Marta Morera Sánchez¹, Fernando Cabrera Naranjo^1^, María del Carmen Pérez Vieitez^1^, Ayoze González Hernández^1^, Jesús Morera Molina^2^, Sara Bisshopp^2^, Pedro Miguel Ponce Marrero^3^

^1^Servicio de Neurología, Hospital Universitario de Gran Canaria Doctor Negrín, 35010 Las Palmas de Gran Canaria, España

^2^Servicio de Neurocirugía, Hospital Universitario de Gran Canaria Doctor Negrín, 35010 Las Palmas de Gran Canaria, España

^3^Servicio de Radiología, Hospital Universitario de Gran Canaria Doctor Negrín, 35010 Las Palmas de Gran Canaria, España

**Objetivos**: Frente al temblor esencial refractario la talamotomía del Núcleo Ventral Intermedio (VIM) mediante Ultrasonido Focalizado de Alta Intensidad (HIFU) guiado por Resonancia Magnética (RM) es una alternativa no invasiva a otras técnicas quirúrgicas. Desde su implantación en el Hospital Universitario de Gran Canaria Doctor Negrín (HUGCDN) hasta abril de 2026 se han realizado 47 procedimientos, con 44 talamotomías efectivas (33 unilaterales, 11 bilaterales). **Material y Métodos**: Estudio observacional descriptivo de pacientes con temblor esencial sometidos a talamotomía HIFU del VIM en el HUGCDN hasta abril de 2026. Se registraron múltiples variables demográficas y clínicas, a destacar la escala Clinical Rating Scale for Tremor (CRST) basal, a 6 y 12 meses, diferenciando entre procedimientos unilaterales y bilaterales secuenciales, así como los efectos adversos. **Resultados**: En las talamotomías unilaterales (n = 33), la edad media fue de 69 años, con 22 hombres (66,7%) y 11 mujeres (33,3%), con un CRST basal de 52, el CRST al año fue de 32, con una mejoría de la calidad de vida a los 6 meses del 67% y una reducción del temblor del 70%. En las bilaterales, la edad media fue de 67 años, con 7 hombres (63,6%) y 4 mujeres (36,4%); el CRST tras el primer tratamiento fue de 36,10 y el CRST post segundo tratamiento de 16,30. **Conclusiones**: La experiencia del HUGCDN demuestra que la talamotomía HIFU es una opción eficaz para el temblor esencial refractario, con una reducción cercana al 70% del temblor y una mejoría relevante de la calidad de vida en los procedimientos unilaterales, y un beneficio adicional significativo en pacientes seleccionados tratados de forma bilateral secuencial.

**CO-02.- Impacto de la Comorbilidad Psiquiátrica en la Respuesta al Tratamiento con Anticuerpos Anti-CGRP en Pacientes con Migraña**

Alicia Auyanet Matejkova^1^, Lucía Bernal González^1^, Juan Francisco García Granado^1^, Yesica Miranda Bacallado^1^, Jorge Cegarra Sánchez^1^, Alejandro Relloso de la Fuente^1^, Marta Morera Sánchez^1^, Ayoze González Hernández^1^, María del Carmen Pérez Vieitez^1^, Fernando Cabrera Naranjo^1^

^1^Servicio de Neurología, Hospital Universitario de Gran Canaria Doctor Negrín, 35010 Las Palmas de Gran Canaria, España

**Objetivos**: La migraña se asocia con frecuencia con comorbilidades psiquiátricas, especialmente ansiedad y trastornos depresivos. El objetivo de este estudio es analizar el impacto de la comorbilidad psiquiátrica en la respuesta al tratamiento con anticuerpos anti-CGRP en una cohorte de pacientes en práctica clínica real. **Material y Métodos**: Estudio observacional, retrospectivo y unicéntrico en una cohorte de 114 pacientes con migraña en tratamiento con anticuerpos anti-CGRP iniciado entre 2020 y 2025. Se recogieron variables demográficas, frecuencia basal de crisis, escalas de impacto (HIT-6 y MIDAS) y presencia de comorbilidad psiquiátrica. La respuesta clínica se evaluó mediante la reducción de días de migraña al mes (DMM) y la variación en las puntuaciones de dichas escalas a los 6 meses. **Resultados**: El 35,5% de los pacientes presentaba comorbilidad psiquiátrica. El tratamiento con anti-CGRP se asoció a una mejoría significativa en todos los outcomes evaluados: reducción del número de DMM (18,45 ± 7,71 a 10,04 ± 8,21; *p* < 0,001), de la puntuación HIT-6 (64,77 ± 5,62 a 57,46 ± 7,60; *p* < 0,001) y de la escala MIDAS (54,43 ± 46,88 a 27,70 ± 32,81; *p* < 0,001). No se observaron diferencias significativas entre pacientes con y sin comorbilidad psiquiátrica en ninguno de los parámetros analizados (*p* > 0,05). **Conclusiones**: Los anticuerpos monoclonales anti-CGRP son eficaces en pacientes con migraña independientemente de la presencia de comorbilidad psiquiátrica. Estos hallazgos sugieren que dicha comorbilidad no condiciona una peor respuesta terapéutica en nuestra muestra.

**CO-03.- Retraso en el Acceso al Código Ictus en Turistas en Gran Canaria: Impacto del Paso por Centros Intermedios**

Leonor Pinta Cóndor^1^, Noemi Bravo Alvarado^1^, Daniel Escáneo Otero^1^, Julián Cruz Funes^1^, Francisco de Asís Suárez Bautista^1^, Arminda Ruano Hernández^1^, Idaira Martín Santana^1^, Raúl Amela Peris^1^

^1^Servicio de Neurología, Complejo Hospitalario Universitario Insular Materno-Infantil (CHUIMI), 35016 Las Palmas de Gran Canaria, España

**Objetivos**: El ictus agudo es una patología tiempo-dependiente en la que el acceso precoz a terapias de reperfusión condiciona el pronóstico. En zonas turísticas, el desconocimiento del circuito sanitario y la asistencia inicial en centros no referentes podrían retrasar el tratamiento. **Material y Métodos**: Estudio observacional retrospectivo de pacientes turistas atendidos como código ictus en el CHUIMI durante 2025. Se excluyeron derivaciones interinsulares. Los pacientes se dividieron en dos grupos: acceso directo al centro de referencia y valoración en centros intermedios. Se recogieron variables demográficas, factores de riesgo, tipo de ictus, tiempos asistenciales, llegada fuera de ventana para Fibrinólisis Intravenosa (FIV), trombectomía y la National Institutes of Health Stroke Scale (NIHSS) inicial. Las variables continuas se expresaron como mediana y rango intercuartílico (RIC). Se emplearon U de Mann-Whitney y test exacto de Fisher. **Resultados**: Se incluyeron 57 pacientes, mediana de edad 70 años; 59,6% fueron varones y el 87,7% presentaron ictus isquémico. El 49,1% fueron valorados previamente en centros intermedios (San Roque y Clínica Roca). La mediana del tiempo desde el inicio de síntomas hasta la llegada al centro de referencia fue mayor en pacientes con paso por centro intermedio frente al acceso directo (383 vs 202 minutos; *p* = 0,129). La llegada fuera de ventana para FIV fue más frecuente en el grupo con centro intermedio (65,4% vs 47,8%; *p* = 0,215). **Conclusiones**: El paso por centros intermedios mostró mayor retraso asistencial y una mayor frecuencia de llegada fuera de la ventana terapéutica en turistas con código ictus. Aunque estas diferencias no alcanzaron significación estadística, podrían representar una barrera asistencial potencialmente modificable.

**CO-04.- Síndrome de Bing-Neel**

Noemi Bravo Alvarado^1^, Leonor Pinta Cóndor^1^, Daniel Escáneo Otero^1^, Francisco de Asís Suárez Bautista^1^, Julián Cruz Funes^1^, Pino López Méndez^1^, Jenifer del Pino Castellano Santana^1^, Ernesto Santana Suárez^1^, Arminda Ruano Hernández^1^, Raúl Amela Peris^1^

^1^Servicio de Neurología, Complejo Hospitalario Universitario Insular Materno-Infantil (CHUIMI), 35016 Las Palmas de Gran Canaria, España

**Objetivos**: El síndrome de Bing-Neel (SBN) es una complicación neurológica extremadamente rara de la macroglobulinemia de Waldenström (MW) producida por infiltración directa del SNC por células linfoplasmocitarias. Su presentación clínica y radiológica es heterogénea, lo que dificulta el diagnóstico. **Material y Métodos**: Varón de 65 años con antecedente de MW que consulta repetidamente por cefalea frontal y episodios fluctuantes de alteración del lenguaje. Posteriormente desarrolla deterioro progresivo de la marcha, caídas, temblor y pérdida ponderal. En la exploración neurológica destacan piramidalismo difuso, dismetría cerebelosa y marcada inestabilidad postural. **Resultados**: Ingresa para estudio realizándose una RM cerebral que evidencia afectación leptomeníngea cerebelosa con lesiones intraaxiales hemisféricas izquierdas, sugestivas de proceso infiltrativo. No se realiza punción lumbar (PL) por posible riesgo de herniación. Ante la sospecha de SBN se inicia tratamiento con zanubrutinib asociado a corticoterapia. El paciente recupera la deambulación y mejora del resto de la clínica. Sin embargo, reingresa el mismo mes por somnolencia y alteración del lenguaje, objetivándose hematoma temporal izquierdo, interpretado como posible complicación hemorrágica secundaria a inhibidores de tirosina quinasa de Bruton (BTKi). Se decide realizar PL donde el líquido cefalorraquídeo (LCR) muestra pleocitosis linfocitaria y componente monoclonal IgM-kappa, compatible con SBN. Ante esta complicación grave, se decide cambio terapéutico a pirtobrutinib, inhibidor no covalente de BTKi. **Conclusiones**: El SBN constituye una complicación excepcional de la MW que requiere alta sospecha diagnóstica. Aunque los BTKi constituyen actualmente el tratamiento de elección, pueden asociarse a complicaciones hemorrágicas potencialmente graves, dificultando aún más el manejo de estos pacientes.

**CO-05.- Experiencia con el Estimulador del Nervio Vago (ENV) en una Consulta Monográfica de Epilepsia**

Julián Cruz Funes^1^, Francisco de Asís Suárez Bautista^1^, Arminda Ruano Hernández^1^, Abel Díaz Díaz^1^, Daniel Cardona Reyes^1^, Idaira Martín Santana^1^, Raúl Amela Peris^1^

^1^Servicio de Neurología, Complejo Hospitalario Universitario Insular Materno-Infantil (CHUIMI), 35016 Las Palmas de Gran Canaria, España

**Objetivos**: La estimulación del nervio vago (ENV) es una intervención terapéutica bien establecida para personas con epilepsia resistente a fármacos. Presentamos los datos correspondientes a los pacientes portadores de ENV en la Consulta de Epilepsia de nuestro Hospital. **Material y Métodos**: Se realizó un estudio observacional que incluyó a pacientes con epilepsia farmacorresistente sometidos a implantación de ENV y en seguimiento en la Consulta de Epilepsia de nuestro centro. Se incluyeron un total de 12 pacientes. La variable principal analizada fue la tasa de respondedores. Asimismo, se recogieron variables clínicas como el tipo de epilepsia, el número de fármacos anticrisis (FACs) previos y actuales, así como la aparición de efectos adversos. **Resultados**: La cohorte incluyó pacientes con síndrome de Lennox-Gastaut, así como epilepsias focales y generalizadas. Nueve de los 12 pacientes se encontraban en tratamiento con más de tres FACs en el momento del análisis, y todos habían recibido al menos cinco FACs a lo largo de su evolución. La tasa de respondedores fue del 66%. Entre los no respondedores, todos experimentaron mejoría en la frecuencia o en la intensidad de las crisis. La media de intensidad del estimulador es de 2,135 mA, en la mayoría de los casos con más de 5 años de seguimiento. Los efectos adversos más frecuentes fueron la sensación de ahogo y la disfonía, de carácter leve. Nueve de los 12 pacientes no presentaron efectos adversos. **Conclusiones**: En nuestra serie, la ENV se mostró como una terapia eficaz y segura en el tratamiento de la epilepsia farmacorresistente. Estos resultados respaldan su consideración como una alternativa terapéutica prometedora y mínimamente invasiva en este grupo de pacientes.

## 2. Comunicaciones Póster

**PO-01.- El Desafío del Diagnóstico Diferencial en el Trastorno Conductual Agudo**

Marta Morera Sánchez^1^, Alicia Auyanet Matejkova^1^, Alejandro Relloso de la Fuente^1^, Jesús de la Nuez González^1^, Juan Francisco García Granado^1^, Jorge Cegarra Sánchez^1^, Fernando Cabrera Naranjo^1^, María del Carmen Pérez Vieitez^1^, Ayoze González Hernández^1^, Santiago Díaz Nicolás^1^

^1^Servicio de Neurología, Hospital Universitario de Gran Canaria Doctor Negrín, 35010 Las Palmas de Gran Canaria, España

**Objetivos**: El trastorno conductual agudo constituye un desafío en neurología por su similitud con trastornos psiquiátricos primarios. Las meningoencefalitis virales, como las causadas por Virus Varicela-Zóster (VVZ) y Virus Herpes Humano (VHH-6), pueden debutar con agitación intensa. La interpretación de la Reacción en Cadena de la Polimerasa (PCR) en LCR es clave, ya que su positividad no siempre indica infección activa, pudiendo corresponder a reactivaciones sin papel patogénico. **Material y Métodos**: Estudio descriptivo de dos casos atendidos en HUGCDN. Se analizaron antecedentes, clínica, LCR (presión, citoquímica, PCR), neuroimagen y respuesta a tratamiento antiviral e inmunomodulador. **Resultados**: • Caso 1: Mujer de 39 años con cefalea y agitación psicomotriz grave. Hipertensión intracraneal, LCR con pleocitosis intensa y PCR positiva para VVZ; RM sin hallazgos. Presentó rápida mejoría tras aciclovir y dexametasona. • Caso 2: Mujer de 74 años con alteración conductual, verborrea y crisis focales. LCR con escasa celularidad y PCR positiva para VHH-6; RM con lesiones corticales en lóbulo frontal derecho, cíngulo e ínsula. La ausencia de respuesta a ganciclovir orientó a mecanismo inmunomediado, con mejoría tras metilprednisolona pese a autoinmunidad negativa. **Conclusiones**: Es importante considerar causas orgánicas en cuadros psiquiátricos agudos, incluso sin signos infecciosos clásicos. La PCR positiva en LCR debe interpretarse con cautela por su posible efecto distractor. La falta de respuesta a antivirales sugiere etiología inmunomediada, donde la corticoterapia precoz es determinante para el pronóstico.

**PO-02.- Talamotomía VIM Mediante HIFU en Temblor Neuropático Refractario: Uso Compasivo en Neuropatía Desmielinizante Severa**

Alejandro Relloso de la Fuente^1^, Juan Diego Guerra Hiraldo^1^, Juan Francisco García Granado^1^, Jorge Cegarra Sánchez^1^, Marta Morera Sánchez^1^, Alicia Auyanet Matejkova^1^, María del Carmen Pérez Vieitez^1^, Fernando Cabrera Naranjo^1^, Ayoze González Hernández^1^, Jesús Morera Molina^2^, Sara Bisshopp^2^, Pedro Miguel Ponce Marrero^3^

^1^Servicio de Neurología, Hospital Universitario de Gran Canaria Doctor Negrín, 35010 Las Palmas de Gran Canaria, España

^2^Servicio de Neurocirugía, Hospital Universitario de Gran Canaria Doctor Negrín, 35010 Las Palmas de Gran Canaria, España

^3^Servicio de Radiología, Hospital Universitario de Gran Canaria Doctor Negrín, 35010 Las Palmas de Gran Canaria, España

**Objetivos**: El temblor neuropático es una forma infrecuente y discapacitante de temblor de acción. Su tratamiento farmacológico suele ser poco eficaz. La evidencia intervencionista es escasa y se limita a casos o pequeñas series con estimulación cerebral profunda del núcleo ventral intermedio talámico (VIM-DBS). A nuestro conocimiento, no existen casos publicados de talamotomía VIM mediante ultrasonido focalizado guiado por resonancia magnética (MRgFUS/HIFU) en esta indicación. **Material y Métodos**: Varón de 78 años con polineuropatía desmielinizante severa en contexto de macroglobulinemia de Waldenström (MYD88 L265P+), biopsia de nervio con pérdida completa de fibras y estudio neurofisiológico compatible con temblor intencional. Presentaba temblor de acción progresivo e incapacitante desde 2020, refractario a rituximab, zanubrutinib, primidona y clonazepam. Situación basal: Clinical Rating Scale for Tremor (CRST) A+B 31; Escala de equilibrio de Berg 6/56. Dado el alto riesgo quirúrgico por comorbilidad cardiovascular y anticoagulación, se realizó talamotomía izquierda del VIM mediante HIFU como uso compasivo. **Resultados**: En la revisión al mes de la intervención, no se registraron efectos adversos. El paciente recuperó parcialmente la capacidad para beber, manipular objetos y pasar páginas. La puntuación CRST en miembro superior derecho mejoró de 11 a 9 puntos, y CRST A+B de 31 a 29. Clinical Global Impression (CGI): 3; Patient Global Impression (PGI): 2. **Conclusiones**: La talamotomía VIM mediante HIFU podría ser una opción viable y segura en casos seleccionados de temblor neuropático refractario, especialmente en pacientes con alto riesgo quirúrgico. Aunque la mejoría objetiva fue modesta, el beneficio funcional percibido y la ausencia de complicaciones apoyan su exploración en esta indicación. Se precisan más casos y seguimiento para definir eficacia, seguridad y perfil de candidatos.

**PO-03.- “Cortical Foot Drop”: Cuando un Déficit Distal Engaña**

Silvia Fregel Frías^1^, Lucas Darío Iacampo Leiva^1^, María José Hernández García^1^, Carolina Madrid Pérez¹, Marcos Millet Oval^1^, Sofía del Águila Romero^1^, Ismael Naim Owrang Calvo^1^, Vanessa Pallarés Santos^1^, Paloma Isabel Dupuy Oria^1^, José Antonio Rojo Aladro^1^

^1^Servicio de Neurología, Complejo Hospitalario Universitario de Canarias, 38320 La Laguna, España

**Objetivos**: La monoparesia distal de miembro inferior suele atribuirse a causas periféricas pero puede tener un origen central. **Material y Métodos**: Caso clínico. **Resultados**: Varón de 47 años, sin antecedentes de interés, trabajador de circo, consulta por debilidad distal de MID de 24h de evolución, súbita, mientras actuaba en zancos. En la exploración destacaba un déficit motor limitado a la flexión dorsal y plantar del pie derecho, hiperreflexia bilateral en miembros inferiores y RCP indiferente. NIHSS 0. La neuroimagen mostró una lesión isquémica aguda frontal izquierda (territorio de la Arteria Cerebral Anterior izquierda), con estudio vascular sin alteraciones. La ecocardiografía transtorácica evidenció un mixoma auricular, por lo que se estableció el diagnóstico de ictus cardioembólico y se derivó a cirugía cardíaca. **Conclusiones**: La debilidad aislada del pie (“cortical foot drop”) puede ser la única manifestación de un ictus cortical en territorio de arteria cerebral anterior (ACA). Aunque puede confundirse con un cuadro periférico, especialmente en pacientes sin factores de riesgo vascular o con antecedente compresivo, se debe solicitar neuroimagen ante la ausencia de un patrón motor anatómico periférico con afectación tanto de la dorsiflexión (nervio peroneo) como de la flexión plantar (nervio tibial anterior) sin afectación sensitiva definida y la presencia de hiperreflexia. La ecocardiografía continúa siendo fundamental, especialmente en pacientes jóvenes sin factores de riesgo cardiovascular (FRCV).

**PO-04.- Cuando el Contraste se Sube a la Cabeza**

Paloma Isabel Dupuy Oria^1^, María José Hernández García^1^, David Alejandro Padilla León^1^, Vanessa Pallarés Santos^1^, Alba Elisa Bartolomé Yumar^1^, Ismael Naim Owrang Calvo^1^, Marcos Millet Oval^1^, Sofía del Águila Romero^1^, José Antonio Rojo Aladro^1^

^1^Servicio de Neurología, Hospital Universitario de Canarias, 38320 La Laguna, España

**Objetivos**: La encefalopatía inducida por contraste (EIC), aunque poco frecuente, es una entidad reconocida producida por el uso de contrastes intravenosos yodados y que puede tomar forma de síndromes neurológicos muy dispares por lo que se hace necesario tener elevada sospecha clínica. **Material y Métodos**: Revisión de dos casos clínicos. **Resultados**: Mujer de 67 años que ingresa en Neumología por cuadro de hemorragia alveolar masiva con necesidad de embolización de arterias bronquiales; durante el postoperatorio comienza con un cuadro de disminución del nivel de consciencia y hemiparesia izquierda que es tratado con dexametasona y levetiracetam con mejoría parcial del déficit motor izquierdo y complicándose durante el ingreso con meningitis y bacteriemia por Listeria Monocytogenes sin relación con el cuadro previo y que requirió ingreso en Unidad de Críticos. Varón de 75 años que requirió angiografía por Tomografía Computarizada (angio-TC) programado para exclusión de aneurisma cerebral diagnosticado tras evento isquémico lacunar el año previo; posterior a la intervención comenzó con crisis comiciales tónico-clónicas generalizadas que se interpretaron como secundarias a la neurotoxicidad por contraste, tratadas de manera conservadora y con recuperación completa. **Conclusiones**: La EIC debe incluirse en nuestro diagnóstico diferencial ante cualquier manifestación neurológica, desde leves hasta graves, pues su diagnóstico es puramente clínico y las implicaciones terapéuticas y pronósticas difieren en gran medida de otras entidades neurológicas.

**PO-05.- Meningitis Tuberculosa (MT) En Paciente Inmunocompetente con Presentación Atípica**

Carolina Madrid Pérez^1^, María José Hernández García^1^, Daniel Rodríguez Díaz^1^, Silvia Fregel Frías^1^, Sofía del Águila Romero^1^, Marcos Millet Oval^1^, Paloma Isabel Dupuy Oria^1^, Vanessa Pallarés Santos^1^, Ismael Naim Owrang Calvo^1^, José Antonio Rojo Aladro^1^

^1^Servicio de Neurología, Complejo Hospitalario Universitario de Canarias, 38320 La Laguna, España

**Objetivos**: La Meningitis Tuberculosa (MT) es una manifestación infrecuente (4%) de la tuberculosis extrapulmonar, con elevada morbimortalidad. **Material y Métodos**: Caso clínico. **Resultados**: Mujer, 69 años, con antecedentes de hipertensión arterial y dislipemia, ingresada por fractura de cadera patológica con infección osteoarticular y varias reintervenciones. Los cultivos estándar son negativos, con cultivo de micobacterias positivo. Tras dos meses de ingreso con buena adherencia a tuberculostáticos, persiste síndrome febril intermitente y episodios confusionales. La paciente presenta cuadro de afasia y hemiparesia derecha súbitas (NIHSS 7) y activan código ictus. La Tomografía Computarizada (TC) y angioTC inicial no muestra lesiones ni oclusiones arteriales, con Hb 5,8 g/dL en análisis urgente. La paciente mejora rápidamente y se atribuye el cuadro a un origen hemodinámico. Posteriormente presenta episodio de desconexión y cefalea, asociando fiebre y somnolencia, por lo que se realiza PL con LCR compatible con MT con pleocitosis linfocitaria, consumo de glucosa, hiperproteinorraquia (763,2 mg/dL) y aumento de Adenosina Desaminasa (ADA) (71,1 U/L), con cultivos negativos. La RM cerebral muestra realce leptomeníngeo y múltiples nódulos extraaxiales supra e infratentoriales, sin isquemia. Se mantuvo tratamiento con tuberculostáticos (rifampicina, isoniacida, pirazinamida y etambutol) y se asoció dexametasona, con mejoría. Tras 8 meses persiste leve empeoramiento cognitivo, con leve inflamación en neuroimagen de control. **Conclusiones**: Los hallazgos en el LCR y la RM cerebral permiten diagnosticar MT, a pesar de los cultivos negativos. La sintomatología neurológica apareció tras meses de tratamiento, por probable reacción paradójica al tratamiento tuberculostático (reconstitución inmune). Debemos pensar en MT en cuadros insidiosos inclusive en inmunocompetentes, dado su gravedad y riesgo de secuelas incluso con tratamiento adecuado.

**PO-06.- Cuando las Carótidas Desaparecen: Adaptación Hemodinámica Cerebral Extrema en Arteritis de Takayasu**

Leonor Pinta Cóndor^1^, Noemi Bravo Alvarado^1^, Daniel Escáneo Otero^1^, Julián Cruz Funes^1^, Francisco de Asís Suárez Bautista^1^, Arminda Ruano Hernández^1^, Idaira Martín Santana^1^, Raúl Amela Peris^1^

^1^Servicio de Neurología, Complejo Hospitalario Universitario Insular Materno-Infantil (CHUIMI), 35016 Las Palmas de Gran Canaria, España

**Objetivos**: La arteritis de Takayasu es una vasculitis crónica de grandes vasos que puede producir estenosis u oclusión progresiva de los troncos supraaórticos, condicionando situaciones hemodinámicas complejas con riesgo de hipoperfusión cerebral. La ecografía Doppler permite valorar de forma dinámica tanto la anatomía vascular como los mecanismos compensatorios del flujo cerebral. **Material y Métodos**: Mujer de 49 años con antecedente en la infancia de episodio autolimitado de hemiparesia izquierda. En la evolución posterior presentó cefalea, episodios presincopales y varios eventos transitorios de debilidad en extremidades de minutos de duración. Tras estudio etiológico fue diagnosticada de arteritis de Takayasu. En revisión neurológica se encontraba sin focalidad. Se realizó ecografía Doppler de troncos supraaórticos y transcraneal, objetivándose ausencia de señal de flujo en ambas arterias carótidas comunes, fenómeno de robo de subclavia con inversión de flujo en arteria vertebral izquierda y perfusión cerebral dependiente predominantemente de la arteria vertebral derecha (se muestran en las imágenes). El estudio transcraneal mostró atenuación difusa de flujos intracraneales. **Conclusiones**: La ecografía Doppler constituye una herramienta fundamental en la valoración funcional de pacientes con arteritis de Takayasu. En este caso permitió identificar una situación de perfusión cerebral críticamente dependiente de una única arteria vertebral funcional, hallazgo de elevada relevancia clínica. El estudio hemodinámico no invasivo resulta esencial para la estratificación de riesgo y seguimiento evolutivo de estos pacientes.

**PO-07.- Síndrome SMART con Resolución Radiológica Completa en Resonancia Magnética de Control a los 3 Meses: A Propósito de un Caso**

Leonor Pinta Cóndor^1^, Noemi Bravo Alvarado^1^, Daniel Escáneo Otero^1^, Julian Cruz Funes^1^, Francisco de Asís Suárez Bautista^1^, Arminda Ruano Hernández^1^, Idaira Martín Santana^1^, Raúl Amela Peris^1^

^1^Servicio de Neurología, Complejo Hospitalario Universitario Insular Materno-Infantil (CHUIMI), 35016 Las Palmas de Gran Canaria, España

**Objetivos**: El síndrome Stroke-like Migraine Attacks after Radiation Therapy (SMART) es una complicación neurológica tardía e infrecuente de la radioterapia craneal. Se caracteriza por episodios subagudos de cefalea tipo migrañosa asociados a déficits neurológicos focales, alteraciones visuales y/o crisis epilépticas, apareciendo años o incluso décadas tras la irradiación cerebral. Su diagnóstico supone un reto clínico, ya que puede simular ictus isquémico, recidiva tumoral, encefalitis o estatus epiléptico focal. La resonancia magnética cerebral es la herramienta diagnóstica clave. La resolución clínico-radiológica en estudios evolutivos apoya firmemente el diagnóstico. **Material y Métodos**: Mujer de 60 años con antecedente de hemangioma radicular en fosa posterior tratado con cirugía y radioterapia en 2004, sin secuelas neurológicas. Consultó por cefalea hemicraneal izquierda de 15 días de evolución, alucinaciones visuales complejas y hemianopsia homónima derecha de inicio reciente. En la exploración destacaban afasia leve y signos corticales dominantes izquierdos. El estudio de LCR, analítica, TC craneal y electroencefalograma (EEG) sin hallazgos relevantes. La resonancia magnética cerebral evidenció lesión córtico-subcortical temporo-parieto-occipital izquierda con hiperintensidad en secuencias FLAIR (fluid-attenuated inversion recovery) y marcado realce giriforme cortical. Ante la sospecha de síndrome SMART se inició tratamiento con metilprednisolona intravenosa, con mejoría clínica progresiva. La resonancia magnética de control a los 3 meses mostró resolución radiológica completa. **Conclusiones**: El síndrome SMART debe considerarse ante episodios neurológicos focales subagudos en pacientes con antecedente de radioterapia craneal. La resonancia magnética cerebral muestra hallazgos característicos y su resolución en estudios de control apoya firmemente el diagnóstico.

**PO-08.- Vino por un Diagnóstico y se le dio Uno y Medio: Dos Posibles Etiologías para una Entidad Rara**

Vanessa Pallarés Santos^1^, Dionisio Miguel García Álvarez^1^, María José Hernández García^1^, Paloma Isabel Dupuy Oria^1^, Ismael Naim Owrang Calvo^1^, Marcos Millet Oval^1^, Sofía del Águila Romero^1^, Carolina Madrid Pérez^1^, Silvia Fregel Frías^1^, José Antonio Rojo Aladro^1^

^1^Servicio de Neurología, Hospital Universitario de Canarias, 38320 La Laguna, España

**Objetivos**: El síndrome del uno y medio de Fisher, consistente en parálisis completa de la mirada horizontal de un ojo (uno), con parálisis de aducción, exotropia y nistagmo de abducción del ojo contralateral (medio); es poco frecuente y se produce por una lesión en la parte dorsal de la protuberancia que afecta a la formación reticular paramediana, fascículo longitudinal medial ipsilateral y frecuentemente núcleo abducens. Puede producirse por distintas etiologías, siendo la vascular más frecuente en mayores de 65. **Material y Métodos**: Caso clínico. **Resultados**: Varón de 70 años, con factores de riesgo cardiovascular, oclusión de arteria carótida interna izquierda y estenosis del 90% de la derecha y enfermedad renal crónica, que acude a urgencias por diplopia binocular y mareo inespecífico. A su llegada, tensión arterial de 185/89 mmHg. A la exploración, presentaba parálisis del ojo derecho para todas las miradas salvo infraversión, con exotropia del ojo izquierdo y limitación a la aducción, llegando hasta línea media sin traspasarla, con nistagmo de abducción. En pruebas de imagen se objetiva una lesión a nivel mesencefálico sugestiva de cavernoma, y una lesión isquémica milimétrica protuberancial aguda parasagital derecha. **Conclusiones**: El síndrome del uno y medio de Fisher es poco frecuente, pero fácilmente reconocible y buen localizador de lesiones en protuberancia. Presentamos un caso representativo, con limitación para la supraversión en el ojo con parálisis completa, explicable por una lesión en mesencéfalo. Inicialmente se sospechó cavernoma hemorrágico, por el riesgo anual de sangrado (0,5–2%). Sin embargo, como malformación vascular, también presenta riesgo de isquemia.

**PO-09.- Fístulas Arteriovenosas Durales Cerebrales: Presentación de un Caso y Revisión de la Literatura**

Sofía del Águila Romero^1^, Cristian Morales Hernández^1^, Marcos Millet Oval^1^, Paloma Isabel Dupuy Oria^1^, Vanessa Pallarés Santos^1^, Alba Elisa Bartolomé Yumar^1^, Ismael Naim Owrang Calvo^1^, José Antonio Rojo Aladro^1^

^1^Servicio de Neurología, Hospital Universitario de Canarias, 38320 La Laguna, España

**Objetivos**: Las fístulas arteriovenosas durales (FAVd) cerebrales son raras y suelen producirse mayormente en la unión de los senos venosos transverso y sigmoide izquierdos. Ocurre en pacientes con antecedentes de trombosis de senos venosos cerebrales, entre otros, y puede presentarse con clínica de hipertensión intracraneal. **Material y Métodos**: Caso clínico. **Resultados**: Varón de 39 años, con AP de fumador activo y trombosis de seno venoso transverso y sigmoide derechos 6 meses antes, de etiología a estudio de forma ambulatoria y con RM cerebral de control a los 4 meses con repermeabilización parcial. Consulta por empeoramiento progresivo de su clínica residual: Alteración visual (miodesopsias y visión borrosa en hemicampo inferior bilateral) y cefalea holocraneal opresiva 1–2 veces/semana con respuesta parcial a Paracetamol. Valorado por Oftalmología, que confirma papiledema, tras lo cual se sospecha fístula dural y se solicita angiografía en la que se confirma la presencia de FAVd tipo 2A Cognard, con aporte arterial de ramas de ambas carótidas externas y vertebrales al seno longitudinal superior y tórcula. **Conclusiones**: Las FAVd con aporte de circulación arterial bilateral y posterior son muy poco prevalentes, se tratan mediante embolización arterial o neurocirugía. Deben ser consideradas siempre cuando haya clínica compatible y antecedentes de trombosis de senos venosos, ya que si no podría complicarse con hemorragias intracraneales.

**PO-10.- Nodopatía Autoinmune por Anticuerpos Anti-Neurofascina 155: Respuesta Clínica a Rituximab en una CIDP Inicialmente Refractaria**

Sofía del Águila Romero^1^, Gabriel Ricardo González Toledo^1^, Marcos Millet Oval^1^, Paloma Isabel Dupuy Oria^1^, Vanessa Pallarés Santos^1^, Silvia Fregel Frías^1^, Carolina Madrid Pérez^1^, Ismael Naim Owrang Calvo^1^, José Antonio Rojo Aladro^1^

^1^Servicio de Neurología, Hospital Universitario de Canarias, 38320 La Laguna, España

**Objetivos**: La polineuropatía desmielinizante inflamatoria crónica (CIDP) engloba un conjunto heterogéneo de neuropatías periféricas inmunomediadas caracterizadas por datos electrofisiológicos de desmielinización y disociación albuminocitológica en LCR. Además de la forma clásica, existen variantes atípicas relacionadas con anticuerpos frente a proteínas del nodo de Ranvier, entre ellas la neurofascina-155. **Material y Métodos**: Caso clínico. **Resultados**: Mujer de 38 años con clínica progresiva durante tres años de polineuropatía sensitivo- motora simétrica, con predominio distal en miembros superiores y proximal-distal en miembros inferiores, asociando temblor postural e importante ataxia de la marcha. El estudio mostró hallazgos compatibles con CIDP: criterios clínicos y neurofisiológicos desmielinizantes, hiperproteinorraquia marcada y engrosamiento con captación de raíces de cauda equina en RM. Los anticuerpos antigangliósidos, anticuerpos contra la glicoproteína asociada a la mielina (anti-MAG) y antineuronales fueron negativos. Presentó escasa respuesta a múltiples ciclos de inmunoglobulinas intravenosas y corticoterapia a altas dosis, desarrollando además importantes efectos secundarios por corticoides. Ante la sospecha de nodopatía autoinmune se solicitaron anticuerpos anti- neurofascina 155, que resultaron positivos, confirmándose el diagnóstico. Se inició tratamiento con Rituximab intravenoso en cuatro dosis semanales, suspendiendo inmunoglobulinas y realizando descenso progresivo de prednisona. Tras el tratamiento presentó mejoría progresiva de la estabilidad, sensibilidad y temblor distal, recuperando parcialmente autonomía funcional para actividades básicas. Persistieron dolor neuropático distal, arreflexia en miembros inferiores y ataxia leve de la marcha. **Conclusiones**: Las nodopatías por anticuerpos anti-neurofascina 155 deben sospecharse en pacientes diagnosticados de CIDP con temblor, ataxia y mala respuesta a terapias convencionales. Su identificación precoz permite plantear tratamientos dirigidos con depleción linfocitaria B, como Rituximab, asociado en nuestro caso a mejoría clínica significativa.

**PO-11.- Enfermedad de Hirayama: Diagnóstico Diferencial de la Debilidad Distal Unilateral**

Sofía del Águila Romero^1^, Gabriel Ricardo González Toledo^1^, Marcos Millet Oval^1^, Paloma Isabel Dupuy Oria^1^, Vanessa Pallarés Santos^1^, Silvia Fregel Frías^1^, Carolina Madrid Pérez^1^, Ismael Naim Owrang Calvo^1^, José Antonio Rojo Aladro^1^

^1^Servicio de Neurología, Hospital Universitario de Canarias, 38320 La Laguna, España

**Objetivos**: La Enfermedad de Hirayama es una mielopatía cervical infrecuente que afecta principalmente a varones jóvenes. Se caracteriza por debilidad y amiotrofia distal unilateral de miembro superior, seguida de estabilización espontánea. Su reconocimiento resulta fundamental para evitar diagnósticos erróneos de enfermedad de motoneurona. **Material y Métodos**: Caso clínico. **Resultados**: Varón de 36 años procedente de Venezuela, remitido a consulta de enfermedades neuromusculares por debilidad y amiotrofia distal de miembro superior izquierdo. Refería inicio a los 16 años, con progresión durante aproximadamente dos años y posterior estabilización clínica espontánea. Negaba dolor u otros síntomas sensitivos en ningún momento. En la exploración neurológica destacaba amiotrofia global de miembro superior izquierdo de predominio distal, especialmente en antebrazo y musculatura intrínseca de la mano, asociando discreta amiotrofia escapular y pectoral ipsilateral. Presentaba debilidad distal de musculatura intrínseca de la mano con preservación de la fuerza proximal. No se objetivaron fasciculaciones ni hiperreflexia. El estudio neurofisiológico mostró patrón neurógeno crónico en miembro superior izquierdo con gradiente distal-proximal compatible con afectación de motoneurona inferior C8-T1. La RM cervical mostraba rectificación cervical y protrusiones discales sin lesiones medulares concluyentes. **Conclusiones**: La enfermedad de Hirayama debe considerarse ante amiotrofia distal unilateral de miembro superior en pacientes jóvenes con evolución autolimitada y ausencia de síntomas sensitivos. La sospecha clínica orienta el estudio neurorradiológico y neurofisiológico adecuado. El reconocimiento precoz de esta entidad permite evitar exploraciones innecesarias y establecer un adecuado diagnóstico diferencial con otras enfermedades de motoneurona.

**PO-12.- Síndrome de Parsonage-Turner Atípico: A Propósito de un Caso**

Marcos Millet Oval^1^, Helena Pérez Pérez^1^, Sofía del Águila Romero^1^, Ismael Naim Owrang Calvo^1^, Vanessa Pallarés Santos^1^, Paloma Isabel Dupuy Oria^1^, Carolina Madrid Pérez^1^, Silvia Fregel Frías^1^, José Antonio Rojo Aladro^1^

^1^Servicio de Neurología, Complejo Hospitalario Universitario de Canarias, 38320 La Laguna, España

**Objetivos**: La Neuralgia Amiotrófica (NA) o síndrome de Parsonage-Turner es una neuritis inflamatoria infrecuente que afecta principalmente a los nervios de la cintura escapular, caracterizada por dolor agudo intenso seguido de debilidad y atrofia muscular. **Material y Métodos**: Caso clínico. **Resultados**: Varón de 29 años, sin antecedentes médicos ni familiares de interés, que acude a Urgencias por debilidad y dolor en brazos. Tras sufrir una infección respiratoria dos semanas antes con fiebre y tos, tratada con amoxicilina e ibuprofeno, presenta importante dolor neuropático (“quemazón, calambre”) en antebrazo izquierdo, hipoestesia en los dos primeros dedos de mano y dificultad progresiva para realizar pinza. Dos semanas después desarrolla síntomas similares en el brazo contralateral. En la exploración presentaba debilidad distal de miembros superiores de predominio izquierdo, hipoestesia en tres primeros dedos y una sutil escápula alada derecha. La RM cerebral, columna y plexo braquial descartó alteraciones estructurales. El análisis de sangre fue normal, incluyendo creatina quinasa (CK), anticuerpos (antigangliósidos y de encefalitis/paraneoplásicos), proteinograma y serologías infecciosas. En LCR, destacó escasa celularidad inflamatoria linfocítica. El electromiograma (EMG) sugirió plexopatía braquial bilateral, predominantemente izquierda, localizada a nivel del cordón lateral, axonal moderada- grave izquierda y leve derecha. Se inició gabapentina 300 mg/8 h, prednisona 60 mg/24 h—2 semanas—y rehabilitación, con mejoría progresiva. **Conclusiones**: La NA presenta una primera fase con dolor intenso agudo, seguido de una segunda fase, días o semanas después, con debilidad parcheada y amiotrofia. Puede haber presentación bilateral (25%). La corticoterapia temprana podría acelerar la recuperación motora y aliviar el dolor, aunque faltan estudios robustos. El 75% presentan recuperación funcional tras 2–3 años.

**PO-13.- Síndrome Cerebeloso Subagudo Progresivo de Inicio Adulto**

Marcos Millet Oval^1^, Zebenzui Mendoza Plasencia^1^, Cristian Morales Hernández^1^, Sofía del Águila Romero^1^, Ismael Naim Owrang Calvo^1^, Paloma Isabel Dupuy Oria^1^, Vanessa Pallarés Santos^1^, Silvia Fregel Frías^1^, Carolina Madrid Pérez^1^, José Antonio Rojo Aladro^1^

^1^Servicio de Neurología, Complejo Hospitalario Universitario de Canarias, 38320 La Laguna, España

**Objetivos**: La ataxia subaguda de rápida progresión debe hacer sospechar una degeneración cerebelosa paraneoplásica (DCP), entidad autoinmune infrecuente y segunda manifestación neurológica paraneoplásica más frecuente. **Material y Métodos**: Caso clínico. **Resultados**: Mujer de 74 años que acude por alteración progresiva de la marcha y caídas de 3 meses de evolución. Antecedentes personales: Diabetes Mellitus tipo 2 (DM2), Dislipemia (DLP), depresión y protrusiones discales cervicales (C4–C7). AF: no reseñables. En la exploración: destaca dismetría izquierda, signo de Stewart-Holmes positivo, marcha atáxica con ampliación de base e imposibilidad para Romberg, con deterioro progresivo. Las neuroimágenes iniciales (TC y RM) no mostraron hallazgos, si bien la RMc de control mostró realce patológico en secuencias T2-FLAIR en cerebelo, estatoacústico, cordón espinal y cauda equina. El electromiograma (EMG) presentaba datos de polineuropatía sensitiva axonal, simétrica y distal de extremidades. En la analítica destacó positividad para anti-Hu en sangre (vitamina B1 y E normales) y en LCR. El TC total-body y la tomografía por emisión de positrones (PET) mostraron adenomegalia paratraqueal derecha, con punción-aspiración con aguja fina (PAAF) positiva para malignidad con factor de transcripción tiroideo 1 (TTF1). Durante su ingreso, se inicia rehabilitación e inmunoglobulinas intravenosas; y tras comentar caso en Comité de Tumores se inicia carboplatino-V16 con mejoría parcial de la marcha. **Conclusiones**: Ante el cuadro comentado, descartada etiología estructural y carencial debe priorizarse la etiología autoinmune/paraneoplásica. La DCP presenta una disfunción pancerebelosa en menos de 3 meses y se debe a la expresión del tumor de proteínas neuronales. El 60–70% de los casos precede al cáncer y, aunque la neuroimagen inicial puede ser normal, la identificación de anticuerpos es fundamental y orienta hacia la búsqueda del tumor (anti-Hu – Cáncer microcítico pulmonar).

**PO-14.- Síndrome de Kleine-Levin: A Propósito de un Caso**

Sandra Rodríguez Martín^1^, Deborah Alonso Modino^1^, Alexis Acosta Brito^1^, Saskia Vigni^1^, Magnamara Oliva Martín^1^, Alejandro Chávez Padrón^1^, Andrea Hernández Chávez^1^

^1^Servicio de Neurología, Hospital Universitario Nuestra Señora de Candelaria, 38010 Santa Cruz de Tenerife, España

**Objetivos**: El Síndrome de Kleine-Levin (KLS) es un trastorno neurológico infrecuente caracterizado por episodios recurrentes de hipersomnia asociados a alteraciones cognitivas y conductuales, con recuperación completa entre crisis. **Material y Métodos**: Se presenta un caso con clínica compatible con KLS, analizando su evolución clínica y el proceso diagnóstico diferencial. **Resultados**: Varón de 15 años, sin antecedentes relevantes, que tras un cuadro gastrointestinal vírico comenzó con apatía y cambios conductuales, inicialmente interpretados como trastorno del ánimo, con resolución espontánea. Un mes después presentó recaída con somnolencia marcada, alteraciones conductuales y del lenguaje. A la exploración destacaban tendencia al sueño, agitación psicomotriz intermitente y lenguaje poco fluido. Las pruebas urgentes (analítica, tóxicos, TC craneal y análisis de LCR), así como los estudios realizados durante el ingreso (RM cerebral, EEG y estudios inmunológicos, infecciosos y oncológicos), resultaron normales. La evolución fue desfavorable, desarrollando intensa hipersomnia, inatención, conducta pueril e hipersexualización. Se administraron megabolos de corticoides y modafinilo, con mejoría clínica. Ante la sospecha de KLS se realizó PET cerebral, que mostró hipometabolismo hipocampal y de la corteza asociativa posterior con hipermetabolismo talámico relativo. Tras el alta inició tratamiento con ácido valproico. Durante el seguimiento presentó nuevos episodios de hipersomnia, motivo por el que se inició litio, con buena tolerancia y ausencia de nuevos brotes hasta la fecha. **Conclusiones**: El KLS debe considerarse ante episodios recurrentes de hipersomnia, hipersexualidad y alteraciones conductuales en adolescentes. Este caso resalta la utilidad del PET cerebral como apoyo diagnóstico y la importancia del reconocimiento precoz para evitar retrasos diagnósticos y pruebas innecesarias.

**PO-15.- Prevalencia y Etiología del Ictus Juvenil en el Área Sanitaria sur de Gran Canaria. Estudio Observacional Retrospectivo**

Daniel Escáneo Otero^1^, Leonor Pinta Cóndor^1^, Noemi Bravo Alvarado^1^, Francisco de Asís Suárez Bautista^1^, Julián Cruz Funes^1^, Idaira Martín Santana^1^, Arminda Ruano Hernández^1^, Raúl Amela Peris^1^

^1^Servicio de Neurología, Complejo Hospitalario Universitario Insular Materno-Infantil (CHUIMI), 35016 Las Palmas de Gran Canaria, España

**Objetivos**: El ictus constituye una de las principales causas de morbimortalidad a nivel mundial. A pesar de los avances diagnósticos y terapéuticos, la incidencia de ictus ha aumentado en los últimos años en población joven. Las causas de este incremento no están claras, aunque se ha relacionado con la mejoría de las técnicas diagnósticas, incremento del consumo de drogas ilícitas y fracaso de las estrategias de prevención primaria. El objetivo de este estudio es describir la prevalencia y etiología del ictus juvenil en nuestra área sanitaria. **Material y Métodos**: Se realizó un estudio observacional retrospectivo incluyendo a pacientes de 18 a 45 años ingresados durante el año 2025 en la Unidad de Ictus de nuestro centro, siendo los criterios de ingreso sospecha de ictus agudo de menos de 24 horas de evolución y situación funcional basal favorable (escala de Rankin modificada ≤2). Se recogieron variables demográficas, tipo de ictus, etiología, consumo de drogas y presencia de foramen oval permeable. **Resultados**: Se incluyeron 20 pacientes (4,4% del total de ingresos): 13 hombres (65%) y 7 mujeres (35%). Diecinueve pacientes (95%) presentaron ictus isquémico y uno (5%) ictus hemorrágico. 3 casos (15%) fueron aterotrombóticos, 3 (15%) cardioembólicos, 2 (10%) microvasculares, 6 (30%) de otras causas determinadas y 5 (25%) criptogénicos. Se identificó Foramen Oval Permeable (FOP) en 7 pacientes (35%) y consumo de drogas en 8 (40%). **Conclusiones**: Los resultados obtenidos son concordantes con los descritos en la evidencia actual sobre ictus juvenil. El patrón etiológico particular en este rango etario, así como la elevada prevalencia de ictus criptogénicos, hacen fundamental el estudio etiológico exhaustivo, incluyendo cribado toxicológico sistemático y búsqueda de causas inhabituales previamente a atribuir causalidad al FOP, especialmente común en este rango etario.

**PO-16.- CADASIL y Esclerosis Múltiple: Un Reto Diagnóstico en el Daño de Sustancia Blanca. Serie de Tres Casos**

Daniel Escáneo Otero^1^, Pino López Méndez^1^, Jenifer del Pino Castellano Santana^1^, Leonor Pinta Cóndor^1^, Noemi Bravo Alvarado^1^, Francisco de Asís Suárez Bautista^1^, Julián Cruz Funes^1^, Idaira Martín Santana^1^, Arminda Ruano Hernández^1^, Raúl Amela Peris^1^

^1^Servicio de Neurología, Complejo Hospitalario Universitario Insular Materno-Infantil (CHUIMI), 35016 Las Palmas de Gran Canaria, España

**Objetivos**: El hallazgo de leucoencefalopatía extensa en neuroimagen puede suponer un importante reto diagnóstico en neurología, especialmente cuando existen hallazgos sugestivos y/o clínica compatible tanto con enfermedad desmielinizante como con microangiopatía hereditaria. Esclerosis múltiple y Arteriopatía cerebral autosómica dominante con infartos subcorticales y leucoencefalopatía (CADASIL) pueden compartir manifestaciones clínicas y radiológicas, dificultando el diagnóstico diferencial e incluso planteando la posible coexistencia de ambas entidades. Presentamos tres casos clínicos ilustrativos valorados en nuestro centro. **Material y Métodos**: Revisión del caso clínico de tres pacientes varones entre la quinta y séptima década de la vida que presentaron leucoencefalopatía confluyente en resonancia magnética cerebral, predominantemente periventricular y temporal anterior bilateral. Se analizaron antecedentes clínicos y familiares, estudios de líquido cefalorraquídeo, estudios genéticos, evolución clínica y radiológica. **Resultados**: Los dos primeros pacientes presentaban diagnóstico previo de esclerosis múltiple de larga evolución por diseminación espacial y temporal, pese a bandas oligoclonales negativas en análisis de líquido cefalorraquídeo. Uno de ellos asociaba migraña. En ambos casos presentaban familiares de primer grado con estudio genético positivo para CADASIL. Se confirmó mutación patogénica en uno de ellos, rechazando el segundo el estudio genético. El tercer paciente presentaba inicialmente sospecha de CADASIL por el patrón radiológico y clínica compatible, con estudio dirigido del gen *NOTCH3* negativo. Posteriormente, la progresión clínica y la positividad de bandas oligoclonales permitieron establecer el diagnóstico de esclerosis múltiple remitente-recurrente con progresión secundaria. **Conclusiones**: La superposición fenotípica y radiológica entre esclerosis múltiple y CADASIL implica un escenario diagnóstico complejo, en el que se deben tener en cuenta la evolución clínica, antecedentes familiares, pruebas de imagen y genéticas para evitar tanto el infradiagnóstico de enfermedades hereditarias como la atribución errónea de lesiones vasculares a enfermedad desmielinizante.

**PO-17.- Enfermedad de Alexander: A Propósito de un Caso**

Francisco de Asís Suárez Bautista^1^, Julián Cruz Funes^1^, Arminda Ruano Hernández^1^, Idaira Martín Santana^1^, Laura Fernández Pérez^1^, Raúl Amela Peris^1^

^1^Servicio de Neurología, Complejo Hospitalario Universitario Insular Materno-Infantil (CHUIMI), 35016 Las Palmas de Gran Canaria, España

**Objetivos**: La enfermedad de Alexander es una leucodistrofia rara causada por mutaciones en el gen *GFAP*, que codifica la proteína glial ácida fibrilar, siendo el único trastorno genético primario de los astrocitos. Presenta una notable heterogeneidad clínica, con formas de inicio infantil, juvenil y adulto. Se describe un caso de enfermedad de Alexander de inicio en la edad adulta y se resalta su relevancia en el diagnóstico diferencial de síndromes atáxicos progresivos. **Material y Métodos**: Mujer de 38 años con antecedentes de caídas frecuentes en la infancia y dificultades de aprendizaje. Presenta un cuadro progresivo de 8 años de evolución con ataxia de la marcha y de miembros superiores, disartria y disfagia. En el último año asocia incontinencia urinaria, estreñimiento, movimientos faciales involuntarios, hipoestesia en miembro inferior izquierdo y despertares asfícticos. Sin antecedentes familiares. En la exploración: disartria escándida, nistagmo multidireccional, mioclonías oromandibulares y esfenopalatinas, hiperreflexia, Romberg positivo, marcha inestable y dismetría. **Resultados**: Estudios de autoinmunidad, LCR, EEG y EMG normales. La RM mostró leucoencefalopatía periventricular simétrica con atrofia del cuerpo calloso, bulbo, cerebelo y médula cervical, y signo de “chipmunk”. El estudio genético confirmó una variante patogénica en *GFAP* (NM_001131019.3:c.262C>T; p.Arg88Cys), de herencia autosómica dominante. **Conclusiones**: La Enfermedad de Alexander es una entidad infrecuente y probablemente infradiagnosticada. Debe considerarse ante síndromes atáxicos progresivos en adultos, especialmente con signos bulbares, piramidales y disautonómicos.

**PO-18.- Galcanezumab en Cefalea en Racimos (CR). Experiencia en Consulta Monográfica**

Francisco de Asís Suárez Bautista^1^, Julián Cruz Funes^1^, Arminda Ruano Hernández^1^, Abián Muñoz García^1^, Idaira Martín Santana^1^, Raúl Amela Peris^1^

^1^Servicio de Neurología, Complejo Hospitalario Universitario Insular Materno-Infantil (CHUIMI), 35016 Las Palmas de Gran Canaria, España

**Objetivos**: La cefalea en racimos (CR) es la cefalea trigémino-autonómica más frecuente. La refractariedad al tratamiento preventivo convencional es habitual en la práctica clínica. El galcanezumab, anticuerpo monoclonal dirigido contra el CGRP (Péptido relacionado con el gen de la calcitonina), ha emergido como una opción terapéutica. Presentamos una serie de pacientes con CR tratados con galcanezumab en nuestro centro. **Material y Métodos**: Se incluyeron 12 pacientes con CR en tratamiento con galcanezumab 240 mg/mes. La variable principal analizada fue la tasa de respondedores definida como reducción de >50% en la frecuencia de las crisis. Se analizan además, el tipo de CR (episódica o crónica), tratamiento concomitante y efectos adversos. **Resultados**: La tasa de respondedores ha sido del 33,33%. Entre los no respondedores, se observó una mejoría parcial en frecuencia o intensidad de las crisis de cefalea. Un paciente llevaba 6 meses de tratamiento, otro 13 meses y el resto más de 2 años. Todos utilizan al menos 2 fármacos preventivos concomitantes. El fármaco presentó una tasa baja de efectos adversos siendo los más frecuentes cansancio, distensión abdominal y reacción cutánea leve. **Conclusiones**: En nuestros pacientes, el galcanezumab mostró eficacia con reducción de frecuencia e intensidad de crisis en pacientes con CR, con buen perfil de tolerabilidad, constituyendo una opción terapéutica en casos de difícil control.

